# Development of camelid single chain antibodies against Shiga toxin type 2 (Stx2) with therapeutic potential against Hemolytic Uremic Syndrome (HUS)

**DOI:** 10.1038/srep24913

**Published:** 2016-04-27

**Authors:** Maria P. Mejías, Yanina Hiriart, Constanza Lauché, Romina J. Fernández-Brando, Romina Pardo, Andrea Bruballa, María V. Ramos, Fernando A. Goldbaum, Marina S. Palermo, Vanesa Zylberman

**Affiliations:** 1 Laboratorio de Patogénesis e Inmunología de Procesos Infecciosos, Instituto de Medicina Experimental, (IMEX), Consejo Nacional de Investigaciones Científicas y Técnicas (CONICET), P. De Melo 3081, Ciudad de Buenos Aires, (C1425AUM), Argentina; 2 INMUNOVA S.A., Av. Patricias Argentinas 435 - Ciudad de Buenos Aires, (C1405BWE), Argentina; 3 Fundación Instituto Leloir, Instituto de Investigaciones Bioquímicas de Buenos Aires–CONICET, Av. Patricias Argentinas 435 - Ciudad de Buenos Aires. (C1405BWE), Argentina

## Abstract

Shiga toxin (Stx)-producing *Escherichia coli* (STEC) infections are implicated in the development of the life-threatening Hemolytic Uremic Syndrome (HUS). Despite the magnitude of the social and economic problems caused by STEC infections, no licensed vaccine or effective therapy is presently available for human use. Single chain antibodies (VHH) produced by camelids exhibit several advantages in comparison with conventional antibodies, making them promising tools for diagnosis and therapy. In the present work, the properties of a recently developed immunogen, which induces high affinity and protective antibodies against Stx type 2 (Stx2), were exploited to develop VHHs with therapeutic potential against HUS. We identified a family of VHHs against the B subunit of Stx2 (Stx2B) that neutralize Stx2 *in vitro* at subnanomolar concentrations. One VHH was selected and was engineered into a trivalent molecule (two copies of anti-Stx2B VHH and one anti-seroalbumin VHH). The resulting molecule presented extended *in vivo* half-life and high therapeutic activity, as demonstrated in three different mouse models of Stx2-toxicity: a single i.v. lethal dose of Stx2, several i.v. incremental doses of Stx2 and intragastrical STEC infection. This simple antitoxin agent should offer new therapeutic options for treating STEC infections to prevent or ameliorate HUS outcome.

Pathogenic Shiga toxin (Stx)-producing *Escherichia coli* (STEC) infections can cause illness with a wide spectrum of severity, from watery diarrhea and hemorrhagic colitis to Hemolytic Uremic Syndrome (HUS), a life-threatening complication^1^. The infection correlates with ingestion of contaminated meat or vegetables, but is also transmitted by water or even person-to-person contact^2^. Sporadic or massive outbreaks have been reported in several developed countries^3^. In other countries, such as in Argentina, HUS shows an endemic behavior and represents a serious public health problem with high morbidity and mortality values^4^.

A striking feature of STEC infections is the production of potent Stxs, responsible for HUS development^5^,^6^. The Stx family is a group of structurally and functionally related exotoxins, that includes toxins produced by *Shigella dysenteriae* serotype 1 and pathogenic *E. coli* strains, which can produce two types of Stx, type 1 (Stx1) and type 2 (Stx2), and their allelic variants. The genes for Stx are encoded by lysogenic lamboid bacteriophages^7^. All Stx have an AB_5_ molecular configuration^8^. An enzymatically active monomeric A subunit, StxA is non-covalently associated with a pentamer of identical B subunits, StxB, responsible for binding to the cell surface receptor globotriaosylceramide (Gb3).

Notwithstanding the magnitude of the social problems caused by STEC infections, no licensed vaccine or effective therapy is presently available for human use. Several groups have developed anti-Stx monoclonal antibodies (mAbs) that have been tested as potential treatments in different animal models of Stx-dependent injury (Reviewed in^9^). Some of these mAbs have also been evaluated in healthy volunteers during phase I studies^10^,^11^. In addition, a phase II study with chimeric monoclonal antibodies against Stx1 and Stx2 is currently taking place in South America, but there are still no conclusive evidence about their therapeutic efficacy^12^,^13^.

In addition to conventional antibodies, members of the Camelid family also produce unusual antibodies that are composed only of heavy chains^14^,^15^. The antigen binding site of these antibodies is composed of one variable domain (VHH). VHH can be expressed as recombinant fragments, and exhibit several valuable characteristics, such as: small size (12–16 kDa), high solubility, high intrinsic stability, easy tailoring into pluripotent constructs (allowing half-life extension strategies), recognition of uncommon or hidden epitopes, low toxicity and ease of manufacture. These properties lead to the development of therapeutic agents in which VHHs outperform other antibody formats^16^,^17^.

The use of VHH-based antitoxin strategies has been previously reported. These VHH-neutralizing agents (VNAs) consist of linked VHHs that bind and neutralize toxin targets, together with an “effector” conventional antibody. VNAs have been developed against botulinum neurotoxin^18^, Stx1 and Stx2^19^, ricin^20^, or *Clostridium difficile* toxins TcdA and TcdB^21^. Recently, it has been shown that inclusion of an albumin-binding peptide prolongs the functional half-life of the VNAs in serum^22^, and the possibility of gene delivery through a recombinant adenovirus, to induce *in vivo* expression of the therapeutic VNAs^22^,^23^.

Considering that Stx2 is the most pathogenic toxin and that blockade of binding to Gb3 should prevent the first step of the toxicity cascade^24^,^25^, we recently developed a novel antigen which comprises the B subunit of Stx2 (Stx2B) fused to the N-terminus of *Brucella* lumazine synthase (BLS)^26^. This highly stable BLS-Stx2B fusion protein proved to be a valuable immunogen for raising high affinity anti-Stx2B antibodies, capable to induce protection in immunized mice and their offspring against i.v Stx2 as well as intragastric STEC intoxication^27^. Therefore, the aim of the present work was to develop recombinant antibodies for therapeutic ends, exploiting the properties of this immunogen to induce high affinity and protective antibodies against Stx2.

Here we report the generation of a family of Stx2B-binding VHHs that neutralize Stx2 *in vitro* at a nanomolar to subnanomolar range. One anti-Stx2B VHH was selected and two copies were fused to one anti-human seroalbumin VHH. This engineered antibody showed increased permanence in circulation and was able to neutralize the *in vivo* effects of Stx2 in three different mouse models of Stx2-toxicity. This novel and simple antitoxin agent should offer new therapeutic options for treating STEC infections to prevent or ameliorate HUS outcome.

## Results

### Selection of anti-Stx2B VHHs

The BLS-Stx2B chimera is a potent immunogen in mice, inducing highly neutralizing anti-Stx2 antibodies^26^,^27^. Thus, we used this immunogen for the development of llama single domain antibodies (VHH). For this purpose, two llamas were immunized four times with BLS-Stx2B chimera. ELISA analyses confirmed a specific antibody response, with anti-Stx2B titers ranging at 1/40,500 (data not shown). VHH Phage display libraries were generated by PCR-amplification of the VHH-repertoire from blood lymphocytes obtained 4 days after the last boost and cloning into the pHEN4 vector, following standard procedures.

Stx2B-specific VHHs were selected by competitive panning of the phage libraries using the immobilized antigen BLS-Stx2B and soluble BLS as a competitor protein. Phage particles specific for Stx2B were successfully enriched. The screening of 200 clones (periplasmic fraction) revealed that 75% of the VHHs were able to bind the toxin in ELISA assays.

Seventy clones with Stx2-binding capacity were sequenced and grouped into 7 families based on amino acid composition and length of the CDR3 (Supplementary Table S1). Over 50% of the clones belonged to family 1, harboring a short CDR3 in comparison with the other analyzed families. Seventeen clones were selected, which included eleven clones belonging to family 1 and one member belonging to each of the remaining families. These antibodies were subcloned, expressed, purified and tested for their *in vitro* neutralizing activity.

**Figure 1:**
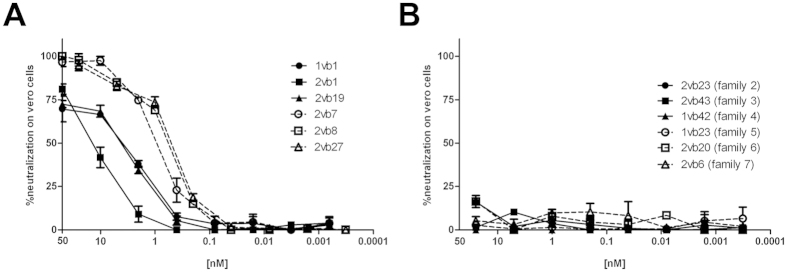
Stx2-neutralizing activity of VHH clones from different families. Serially diluted purified VHHs were tested on the Vero cell neutralization assay as detailed in Materials and Methods. The percentage of cell survival, which is a measurement of the toxin neutralization, was calculated by the following formula: [(OD_toxin+VHH_ − OD_toxin only_)/(OD_no toxin_ − OD_toxin only_)] × 100. Each sample was tested by triplicate and is represented as mean ± SEM. (**A**) VHH clones from family 1. Six representative clones from family 1 are shown. (**B**) VHH clones from other families. One representative clone from each family is shown.

Serial dilutions of all VHH were tested on the Vero cell cytotoxic assay. The neutralization results showed that all members of family 1 were able to neutralize Stx2 cytotoxicity, although with slight differences in their potency (Fig. 1A shows 6 representative clones of family 1). No neutralizing capacity was found in any of the other analyzed families (Fig. 1B).

Since the aim of this work was to develop a therapeutic antibody for clinical use, we selected the yields of recombinant expression and the similarity to human VH sequences as criteria to facilitate further development. In this regard, from the 11 clones belonging to family l, we selected VHH 2vb27 because it showed the highest level of expression (data not shown) and the best developability sequence for production and humanization.

### Multimeric formatting of VHH 2vb27

In order to improve the *in vivo* serum persistence and the avidity of VHH 2vb27, bivalent and trivalent formats of this molecule were performed. The bivalent format [(2vb27)_2_] comprises two copies of 2vb27. For the trivalent format [(2vb27)_2_-SA], a VHH with affinity for human and mouse serum albumin (SA)^28^ was attached to two copies of VHH 2vb27, one through its N-terminal end and other through its C-terminal end (Supplementary Fig. S1).

**Figure 2:**
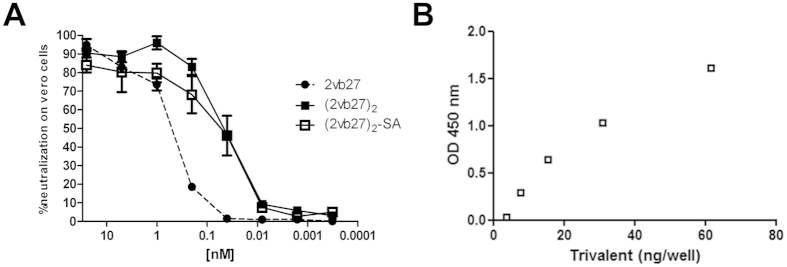
*In vitro* activity of 2vb27 VHH under different formats. (**A**) Serially diluted purified 2vb27, (2vb27)_2_ or (2vb27)_2_-SA were tested on the Vero cell neutralization assay as detailed in Materials and Methods. Each sample was tested by quadruplicate and is represented as mean ± SEM. (**B**) Binding of (2vb27)_2_-SA to human seroalbumin (SA). Nickel coated Maxisorp microtiter plates were incubated with serially diluted (2vb27)_2_-SA. Biotinylated human SA was added and binding was detected with Streptavidin-HRP. Reaction was developed with TMB and absorbance was read at 450 nm.

Since neutralization activity against the whole Stx2 toxin is central to provide protection, (2vb27)_2_ and (2vb27)_2_-SA were tested in the Vero cell cytotoxicity assay and were compared with its parental VHH 2vb27. Figure 2A shows dilution curves for the three molecules from which the IC_50_ was calculated (mean IC_50_ ± SD): 2vb27: 0.5 ± 0.13, (2vb27)_2_: 0.04 ± 0.007***, (2vb27)_2_-SA: 0.06 ± 0.04***; ***p < 0.001 vs 2vb27 (ANOVA). Both (2vb27)_2_ and (2vb27)_2_-SA showed a higher neutralizing activity than its parental VHH 2vb27, indicating that the engineered antibody increased the *in vitro* neutralizing capacity. We also tested the ability of (2vb27)_2_-SA to bind human SA using an ELISA assay (Fig. 2B). Figure 2B shows that (2vb27)_2_-SA was able to bind human SA indicating that both, neutralization activity and binding to SA, were not affected in the fusion protein.

### *In vivo* activity of VHH 2vb27 under different formats

**Figure 3:**
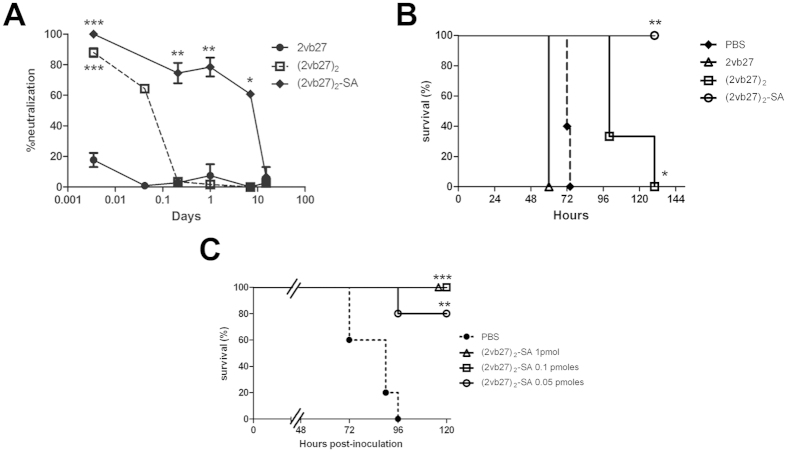
*In vivo* activity of 2vb27 VHH under different formats. (**A**) Persistence in circulation of VHHs. Naïve mice (4 mice/group) were inoculated with 0.5 nmoles of 2vb27, (2vb27)_2_ or (2vb27)_2_-SA, and they were subsequently bled at the indicated time points. *In vitro* Stx2-neutralizing capacity of plasma samples (dilution 1/50) was measured on Vero cells as described in Materials and Methods. Each sample was tested by triplicate and is represented as mean ± SEM. ***p < 0.001 vs 2vb27, **p < 0.01 and *p < 0.05 vs 2vb27 and (2vb27)_2_ at the same time points. ANOVA. (**B**) *In vivo* Stx2-neutralizing activity of VHHs. Naïve mice (3–5 mice/group) were injected i.v. with 1LD_100_ (0.05 pmoles/mouse) of rStx2, and simultaneously injected with 100 μl of PBS, 33 pmoles of 2vb27, 100 pmoles (2vb27)_2_ or 10 pmoles of (2vb27)_2_-SA. *p < 0.05 and **p < 0.01, vs 2vb27 and PBS groups. Log-Rank test. (**C**) Minimal effective dose of (2vb27)_2_-SA. Naïve mice (5–6 mice/group) were injected i.v. with 1LD_100_ (0.05 pmoles/mouse) of rStx2 and simultaneously injected with 100 μl of PBS (control) or with 1, 0.1 or 0.05 pmoles of (2vb27)_2_-SA. Animals were observed three times a day and survival was recorded. **p < 0.005 and ***p < 0.001 vs PBS, Log-Rank test.

An important property of a therapeutic antibody is the time that it remains in circulation conserving its biological activity. Thus, we first assessed the *in vivo* half-life of VHH 2vb27 under the three different formats, by inoculating naïve mice with 0.5 nmoles of each molecule, and bleeding them subsequently at different time points. The remaining VHH in circulation was evaluated by *in vitro* Stx2-neutralizing activity in plasma. The *in vivo* half-life studies showed that most of VHH 2vb27 was removed within 5 minutes from plasma, (2vb27)_2_ within 5 hours and (2vb27)_2_-SA within 15 days (Fig. 3A).

In addition, 2vb27, (2vb27)_2_ and (2vb27)_2_-SA were directly assessed for their *in vivo* Stx2-neutralization potency. Naïve adult BALB/c mice were i.v. injected with 1LD_100_ of rStx2 (0.05 pmoles/mouse). Simultaneously, they were i.v. injected with each of the three 2vb27 VHH formats. Figure 3B shows that, while (2vb27)_2_-SA fully protected mice against the Stx2-lethal dose, (2vb27)_2_ was only able to confer a delay in the time of death, even injected at a 10-fold higher concentration. On the other hand, monomeric 2vb27 was unable to give any protection against a lethal dose of Stx2. This lack of protection may be due to its low half-life in circulation. Moreover, the higher Stx2 protective capacity of (2vb27)_2_-SA compared to (2vb27)_2_ could be related to the higher *in vivo* half-life, and/or a different bio-distribution through its seroalbumin binding ability.

### Minimal protective dose of (2vb27)_2_-SA

Since (2vb27)_2_-SA showed the highest *in vivo* Stx2 neutralizing activity, we continued the protective studies to determine the minimal effective dose with this format. Naïve adult BALB/c mice were i.v. injected with 1LD_100_. Simultaneously, they were i.v. injected with 100 μl of PBS or 1 pmol, 0.1 or 0.05 pmoles of (2vb27)_2_-SA. Figure 3C shows that up to 0.1 pmoles of (2vb27)_2_-SA were able to fully protect mice against the challenge, while 0.05 pmoles were able to protect 80% of mice.

### Protective capacity of (2vb27)_2_-SA in the mouse model of incremental doses of rStx2

**Figure 4:**
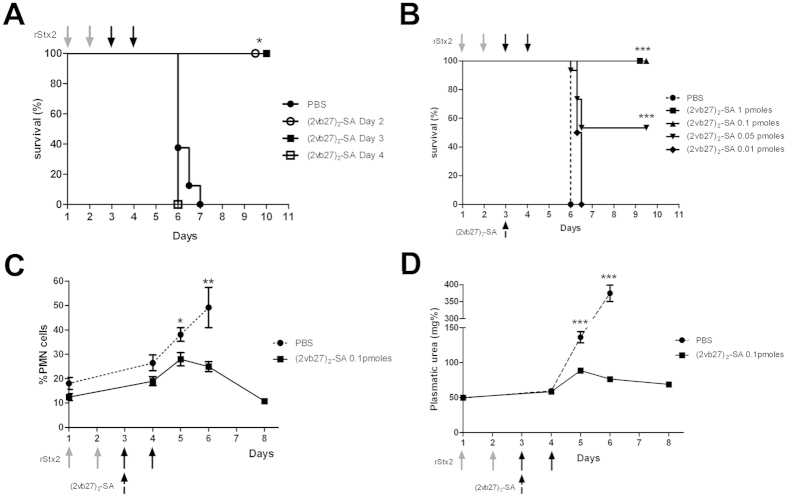
*In vivo* protective capacity of (2vb27)_2_-SA in the incremental split-dose model of Stx2 intoxication. One LD_100_ of rStx2 (0.05 pmoles/mouse) was divided in four consecutive daily doses (the first two doses were 0.009 pmoles/mouse, grey arrows; and the last two doses were 0.016 pmoles/mouse, black arrows). (**A**) Therapeutic window. Mice (4–6 mice/group) were injected with the split dose model and with (2vb27)_2_-SA at day 2, or day 3, or day 4. *p < 0.05 vs PBS, Log-Rank test. (**B**) Lowest protective dose. Mice were injected with the split-dose model and treated with different doses [1 pmoles (n = 3), 0.1 pmoles (n = 6), 0.05 pmoles (n = 15) or 0.01 pmoles (n = 6)] of (2vb27)_2_-SA on day 3 (dashed arrow). Mice were injected with 100 μl of PBS as control (n = 12). ***p < 0.0001 vs PBS, Log-Rank test. (**C,D**) Systemic signs of Stx2-associated toxicity. Mice were injected with the split-dose model and treated with 0.1 pmoles of (2vb27)_2_-SA on day 3. Each time point represents the mean ± SEM for 6–8 mice/group. (**C**) Relative number of PMN cells. *p < 0.05, **p < 0.01 vs (2vb27)_2_-SA. Student t test. (**D**) Plasma urea levels. ***p < 0.001 vs (2vb27)_2_-SA. Student t test.

In addition, we tested (2vb27)_2_-SA in a mouse model of incremental doses of Stx2 that we have developed in our laboratory, because it resembles more closely the human pathologic situation and allows evaluation of the maximal time frame in which treatment should be administrated in order to be effective. For this model, the lethal dose of rStx2 was divided in four doses that were i.v. administrated to naïve adult BALB/c mice, once a day for 4 consecutive days. Trivalent (2vb27)_2_-SA was i.v. administered as a unique high dose (10 pmoles) at different time points during the intoxication protocol. Treatment of mice with only one dose of (2vb27)_2_-SA on days 2 or 3 completely protected them against the acute lethal Stx2-toxicity. In contrast, mice that were treated with (2vb27)_2_-SA on day 4 did not survive (Fig. 4A). In order to determine the lowest therapeutic dose of (2vb27)_2_-SA in this model, different amounts of (2vb27)_2_-SA were administered on day 3 (the last time point in which protection was observed). Mice were i.v injected with 1 pmoles, 0.1 pmoles, 0.05 pmoles or 0.01 pmoles of (2vb27)_2_-SA. Mice were injected with 100 μl of PBS as control. Figure 4B shows that 0.1 pmoles of (2vb27)_2_-SA was the lowest dose to induce a total protection against the Stx2-lethal effect, while 0.05 pmoles partially protected (50%) and 0.01 pmoles did not protect mice.

### Evaluation of Stx2 associated damage in the mouse model of incremental doses of rStx2

In order to assay a more sensitive parameter for Stx2-damage than lethality, we evaluated clinical parameters associated to Stx2 toxicity, particularly neutrophilia and elevated plasmatic urea^29^. Figure 4C,D show that mice treated with the split-dose model and 0.1 pmoles of (2vb27)_2_-SA at day 3 showed a slight and transitory increase in the %PMN cells and in plasmatic urea at day 5 but these values returned to normal by day 8. In contrast, mice receiving no VHH treatment presented increasing levels of %PMN and plasmatic urea up to death (Fig. 4C,D).

### Protective capacity of (2vb27)_2_-SA in the mouse model of intragastric STEC infection

**Figure 5:**
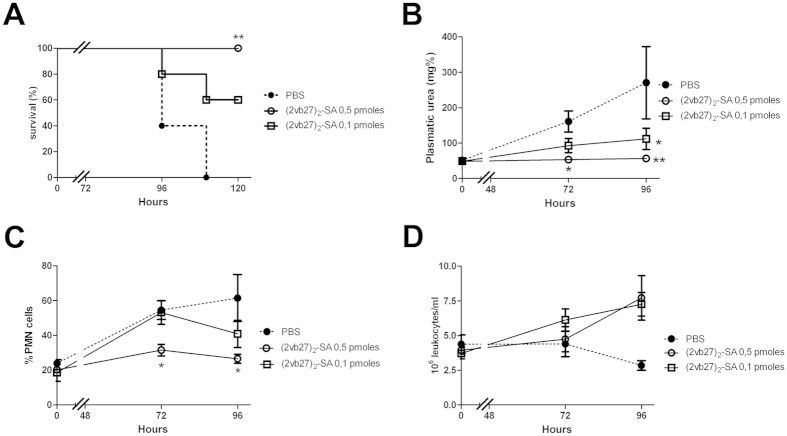
*In vivo* protective capacity of (2vb27)_2_-SA against STEC-induced pathogenicity. (**A**) Survival rates in response to a lethal intragastric (i.g.) challenge with STEC. Seventeen- to nineteen-day-old mice (n = 5–6 mice/group) were injected i.p. with 50 μl of PBS, 0.5 pmoles of (2vb27)_2_-SA or 0.1 pmoles of (2vb27)_2_-SA. Immediately after injection, mice were infected i.g. with 4 × 10^11^ CFU/kg of Stx2-producing *E. coli* O157:H7. **p < 0.005 vs PBS. Log-Rank test. (**B**) Renal Stx2-induced toxicity. Plasmatic urea levels at 72 and 96 h post-STEC challenge were measured as a correlate of renal damage. Each time point represents the mean ± SEM of 5–6 mice/group. *p < 0.05 and **p < 0.01 vs PBS at the same time points. ANOVA. (**C,D**) Systemic signs of Stx2 toxicity. Mice were bled at 72 and 96 h post-STEC challenge, and total and differential counts of leukocytes were assayed. Each time point represents the mean ± SEM from 5–6 mice/group. (**C**) Relative number of PMN cells. *p < 0.05 vs PBS at the same time points. ANOVA. (**D**) Total number of leukocytes.

In order to demonstrate the efficacy of a new therapeutic agent, it is important to test it in several and different animal models. The weaned mouse model has proven to be a reliable animal model for studying the pathology of STEC infection^30^,^31^. We demonstrated that weaned mice infected with Stx2-producing *E. coli* O157:H7 strains develop renal dysfunction and die during the following three-four days after infection. In the same study we also reported that a similar infection with a non-Stx2-producing *E. coli* O157:H7 strain does not result in any of the pathological changes seen with the Stx2-producing strain^32^. Hence, in this animal model, all of the described pathological changes are Stx2-dependent. For these reasons, we chose the weaned mouse model to assay the capacity of (2vb27)_2_-SA to protect mice from lethality induced by STEC infection. Naïve weaned mice were i.p. injected with PBS or 0.5 or 0.1 pmoles of (2vb27)_2_-SA immediately before being challenged with 4 × 10^11^ CFU/kg of Stx2-producing *E. coli* O157:H7. Animals were observed daily and blood was collected until death. Figure 5A shows that while mice receiving PBS died, those receiving 0.5 pmoles of (2vb27)_2_-SA were totally protected against lethality, and those receiving 0.1 pmoles showed a protection of 50%. Mice treated with PBS presented high levels of plasmatic urea previous to death, indicating renal dysfunction (Fig. 5B). In addition, these mice presented an increased percentage of circulating neutrophils and leukopenia, other signs of Stx2-associated poor evolution that have been previously described^32^ (Fig. 5C,D). In contrast, mice treated with 0.5 pmoles of (2vb27)_2_-SA showed normal values of plasmatic urea, circulating neutrophils and leukocytes. Interestingly, mice treated with 0.1 pmoles of (2vb27)_2_-SA presented an intermediate clinical evolution between these two groups (Fig. 5C,D).

### Involvement of the reticuloendothelial system in protection against Stx2 by (2vb27)_2_-SA

**Figure 6:**
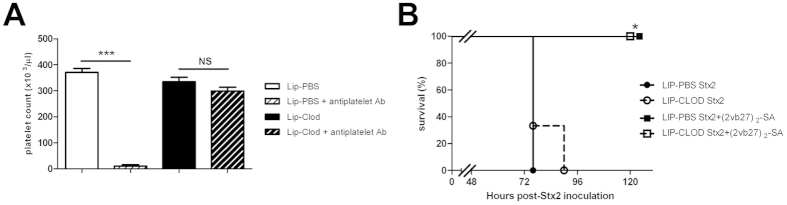
Involvement of the reticuloendothelial system in protection against Stx2 by (2vb27)_2_-SA. (**A**) Depletion of splenic macrophages by Lip-Clod treatment. Adult BALB/c mice (3–4 mice/group) were injected i.v. with a unique dose of 200 μl of Lip-Clod or Lip-PBS. Twenty-four hours later, the animals were injected i.p. with 25 μl (diluted 1:4 in PBS) of a rabbit anti-mouse platelet antiserum. Five hours after antibody inoculation, mice were bled and platelets were counted in a veterinary automatic hematology analyzer. ***p < 0.001. NS: not significant. ANOVA. (**B**) Protection of (2vb27)_2_-SA against rStx2 in the absence of splenic macrophages. Adult BALB/c mice (3 mice/group) were i.v. injected with of 200 μl of Lip-Clod or Lip-PBS. Twenty-four hours later, mice were i.v. injected with 1LD_100_ of rStx2 pre-incubated 1 h at 37 °C with or without 1 pmol/mouse of (2vb27)_2_-SA. *p < 0.05 vs Lip-PBS Stx2 and Lip-Clod Stx2. Log-Rank Test.

To determine whether clearance of Stx2:(2vb27)_2_-SA complexes by the reticuloendothelial system is necessary for protection, mice were depleted of hepatic and splenic macrophages by administration of liposome-encapsulated clodronate (Lip-Clod). The complete disruption of the antibody-dependent clearance by Lip-Clod was corroborated in parallel testing platelet counts in mice injected with anti-platelet antibodies (Fig. 6A). Twenty-four hours after Lip-Clod treatment, mice were i.v. injected with 1LD_100_ of Stx2 pre-incubated with or without (2vb27)_2_-SA. Results shown in Fig. 6B indicate that when the antibody-dependent clearance system is halted, protection against Stx2 by (2vb27)_2_-SA treatment is not affected. These results allow us to conclude that a functional reticuloendothelial system was not necessary during (2vb27)_2_-SA protection against Stx2 toxicity.

## Discussion

The need for specific therapeutic tools against HUS became evident during the last outbreak of *E. coli* O104:H4 in Germany in 2011^3^. Despite several experimental Stx-neutralizing approaches (Reviewed in^9^) and a phase II study with conventional anti-Stx1/2 monoclonal antibodies^12^,^13^, no effective specific therapy is currently available for human use. Although STEC can produce Stx1, Stx2 or both, HUS is most frequently observed in patients undergoing infections with Stx2-producing bacteria^33^. Therefore, Stx2-based therapies are preferred over Stx1. In particular, antibodies against the B subunit of Stx2 (Stx2B) are desirable because interfering with the binding of Stx2 to its receptor Gb3 on the cell surface would block entry of the toxin to the cell and thus, the cytotoxicity cascade. Therefore, the anti-Stx2 therapeutic effectiveness of the antibody would be based on the capacity to efficiently interfere with Stx2-Gb3 interaction and to induce a rapid Stx2-clearance. In this regard, and because two of the three Gb3 binding sites are formed by residues contributed by neighboring monomers^25^, we hypothesized that specific antibodies against conformational epitopes that are located mostly at the interfaces between adjacent monomers would be important to interfere with Gb3 binding. Thus, an adequate immunogen is mandatory to generate protective antibodies. We have recently developed a chimeric protein which comprises a monomer of Stx2B fused to the N-terminus of a monomer of *Brucella* lumazine synthase (BLS, BLS-Stx2B). In such chimeric protein, the conformational epitopes in Stx2B are stabilized, resulting in an efficient immunogen that raises highly Stx2-neutralizing antibodies^26^,^27^.

Moreover, single domain camelid antibodies are prone to recognize conformational epitopes into crevices of proteins, in contrast with conventional antibodies that recognize mostly planar epitopes. Thus, we reasoned that they would be ideal for neutralization of the Gb3-binding activity of Stx2. Therefore, we immunized llamas with BLS-Stx2B and selected VHH 2vb27, which proved to be an excellent candidate to develop a therapeutic agent due to its high expression levels, its developability profile and most important, its high neutralizing activity.

Since the molecular weight cutoff for glomerular filtration is thought to be 30–50 kDa^34^, VHHs (≅15 kDa) are rapidly cleared from circulation. Although this is advantageous in some cases, for example in targeting of VHHs coupled to toxic substances to tumors or in *in vivo* diagnosis using imaging, for other therapeutic applications the short serum half-life of less than an hour^35^ is a disadvantage. Improvement of the pharmacokinetic and dynamic properties of the desired VHH can be achieved by fusion to a second VHH targeted to long-lived serum proteins such as albumin^36^,^37^ or immunoglobin^38^.

Therefore, two molecules were designed: a bivalent format consisting of two copies of 2vb27 [(2vb27)_2_] and a trivalent format consisting of two copies of 2vb27 linked to a VHH recognizing mice/human seroalbumin [(2vb27)_2_-SA]^28^. Fusion of 2vb27 to an anti-albumin VHH highly increased the serum persistence: while VHH 2vb27 was removed from serum within 5 minutes and (2vb27)_2_ within 5 hours, (2vb27)_2_-SA was removed from serum within 15 days.

In addition, *in vitro* studies showed a higher neutralization capacity of (2vb27)_2_ and (2vb27)_2_-SA in comparison with the parental VHH 2vb27. This may be explained by an increased avidity for the antigen, due to the higher number of possible VHH:antigen interactions. Although both (2vb27)_2_ and (2vb27)_2_-SA showed increased *in vitro* capacity, (2vb27)_2_-SA was significantly more effective to protect *in vivo* against Stx2 toxicity than (2vb27)_2_. In fact, linking the anti-Stx2B VHHs to an anti-albumin VHH increased more than 1000 fold the *in vivo* antitoxin potency. Thus, while 100 pmoles of (2vb27)_2_ only delayed the lethal Stx2–effect, 0.1 pmoles of (2vb27)_2_-SA protected 100% of mice intoxicated with 1LD_100_ of Stx2 administered as a single dose or as split-doses over four consecutive days. Moreover, 0.5 pmoles of (2vb27)_2_-SA completely protected mice against a lethal STEC intragastric infection. Thus, (2vb27)_2_-SA proved to be extremely potent and long lasting, displaying a high *in vivo* efficacy. This fact not only highlights the crucial role of the half-life of therapeutic molecules for their effectiveness, but also that binding to mouse seroalbumin could positively affect the biodistribution of the antibody to the sites where the toxin is present *in vivo*.

Furthermore, the mouse model of incremental doses of Stx2 demonstrated that administration of (2vb27)_2_-SA after Stx2-associated clinical signs had already started, protected mice against lethality and restored leukocyte counts and renal parameters to normal values. Thus, these results highlight two key aspects of this molecule as a promising specific treatment: 1) even when partial Stx2-dependent damage had occurred, administration of (2vb27)_2_-SA was effective to stop the bad clinical evolution and reverse the systemic signs of toxicity; 2) because of the long serum persistence of (2vb27)_2_-SA, a low single dose was able to completely block the Stx2 that entered systemic circulation even after therapy administration. Since children usually have clinic signs of Stx-associated toxicity during a time frame in which STEC could still be colonizing the intestine and consequently could be releasing Stx to the bloodstream, these two characteristics are very important and valuable in a potential therapeutic molecule.

The VHH-based molecule presented herein overcomes previously anti-Stx antibodies^9^,^19^ in several features. In particular, Tremblay *et al.* showed that the addition of an “effector” mAb was necessary for protection of their VNAs^19^. In contrast, the trivalent (2vb27)_2_-SA molecule *per se* is highly effective in providing *in vivo* protection in three different Stx2-dependent injury models. This fact may be related to the quality of the monomeric VHHs, underlining the importance in the selection of the immunogen during the development of protective antibodies, and/or the coupling with the anti-seroalbumin VHH. In addition, (2vb27)_2_-SA did not require a conventional mAb to be highly protective, leading to two advantageous consequences: 1) it avoids Fc-dependent cellular interactions and subsequently undesired side-effects derived from these interactions and 2) the commercial development is simpler and less expensive compared to the strategy requiring the addition of a conventional mAb.

Considering the absence of an Fc fragment in (2vb27)_2_-SA, it is not clear if reticulo-endothelial dependent clearance is required to promote the depuration of toxin from serum. Thus, we evaluated the efficacy of (2vb27)_2_-SA to protect mice when the reticulo-endothelial system was completely abrogated by Lip-Clod treatment. These studies indicated that macrophagic clearance is not necessary for the efficacy of (2vb27)_2_-SA. Possibly, neutralized Stx2 in circulation is degraded by the proteolytic activity of serum before interacting with its receptor on target cells, or the (2vb27)_2_-SA:Stx2 complexes are distributed in tissues, due to their seroalbumin-binding property. Moreover, the lack of clearance of (2vb27)_2_-SA:Stx2 complexes by the reticulo-endothelial system is an additional benefit of these antibodies in comparison to conventional mAbs and the previously described VNAs, in which the “effector” mAb was necessary to confer *in vivo* protection, presumably by driving clearance of Stx-VHH immunecomplexes from serum to liver^18^,^19^,^39^. Since clearance of small immune-complexes occurs in the liver, presumably by low affinity Fc receptor-mediated endocytosis primarily in Kupffer cells^39^,^40^, the endocytosis of immune-complexes containing ribosomal toxins such as Shiga toxin could lead to cell death.

In summary, our results reinforce the importance of the design of anti-Stx2 therapeutic agents, taking into account the affinity, half-life and biodistribution in order to achieve a high protective efficacy. More important, they demonstrate that this simple VHH-based antitoxin agent constitutes a potent new therapeutic tool for treating STEC infections even when patients have shown Stx-associated clinical signs, in order to prevent or ameliorate HUS outcome.

## Methods

### Animals

BALB/c mice were bred in the animal facilities of the Instituto de Medicina Experimental (IMEX), Buenos Aires. Experiments performed herein were approved by the IMEX Animal Care Committee and were carried out in accordance with the principles set forth in the Guide for the Care and Use of Laboratory Animals^41^. Throughout these studies, the health and behavior of the mice were assessed three times a day. Any mice that became moribund were humanely euthanized in CO_2_ chamber. IMEX Animal Care Committee guidelines were used to define humane endpoints.

Llamas (*Lama glama*) were bred in the Instituto Nacional de Tecnología Agropecuaria (INTA), Buenos Aires. Experiments performed herein were approved by the INTA animal care committee (*Comité Institucional para el Cuidado y Uso de Animales de Experimentación-CICUAE*) and were conducted in accordance with the approved guidelines.

### Protein production and llama immunizations

BLS-Stx2B was produced and purified as previously described^26^. Two llamas were immunized four times, with a time interval of 15 days, with 0.1 mg/dose of BLS-Stx2B emulsified in Incomplete Freund’s adjuvant by intramuscular injection. The humoral immune response was monitored in serially diluted sera by indirect ELISA using BLS-Stx2B as antigen. Animals were bled 4 and 8 days after the last boost.

### Construction of phage display libraries

Peripheral blood lymphocytes were isolated from 150 ml of blood from each llama by Ficoll-PaqueTM (GE Healthcare, Chalfont St Giles, UK) gradient centrifugation. Total RNA was purified by RNeasy Midi kit (Qiagen, Valencia, CA) and subjected to cDNA synthesis with oligo-dT primers. VHH-encoding regions were amplified by nested PCR^42^. The first PCR was performed with primers Forward (5′-GTCCTGGCTGCTCTTCTACAAGG-3′) and Reverse (5′-GGTACGTGCTGTTGAACTGTTCC-3′). Forward primer anneals to the leader signal sequence of both, VH and VHH. Reverse primer is a camelid IgG CH2-specific primer. Fragments of approximately 700 bp were purified by Qiagen gel extraction kit and used as template for a second PCR with primers VHH-For (5′-GGACTAGTGCGGCCGCTGGAGACGGTGACCTGGGT-3′) and VHH-Back (5′-GATGTGCAGCTGCAGGAGTCTGGRGGAGG-3′, R is A or G) as previously described^42^. PCR products were purified, digested with PstI and NotI (Roche Life Science, Indianapolis, Indiana) and cloned into the pHEN4 phagemid vector for phage display^43^. Transformation into TG1 *E. coli* (Lucigen, Middleton, WI) yielded libraries with sizes of 10^8^ clones.

### Panning and screening for selection of Stx2B-specific VHHs

Phage particles were precipitated with polyethylene glycol from culture supernatants of transformants infected with M13K07 helper phage (Invitrogen, Camarillo, CA). Panning of specific phages was performed using BLS-Stx2B immobilized on Maxisorp microtiter plates (Nunc, Thermo Fisher Scientific, Waltham, MA), blocked with casein 0.1% in PBS and washed 20 times with PBS-Tween-20 0.05% solution with 1 μM BLS. After washing, bound phages were eluted using 0.1 M trimethylamine (TEA) pH 10, followed by neutralization with 1 M Tris pH 7.4. The enrichment of Stx2B-specific phages was monitored by phage titration, after infection of TG1 *E. coli* cells. Individual clones were picked, grown in 24-well plates at 37 °C and the VHH production was induced with 1 mM IPTG when bacteria reached log-phase overnight. After production, the periplasmic fractions were recovered and tested on Maxisorp microtiter plates coated overnight at 4 °C with BLS-Stx2B or BLS (100 ng/well) and blocked with 3% PBS skimmed milk. Rat monoclonal anti-HA peroxidase conjugated Antibody (recognizes the HA peptide sequence YPYDVPDYA derived from the human influenza virus hemagglutinin protein; Roche) was used for detection. Reaction was developed with O-phenylenediamine (Sigma, St Louis, MO) and absorbance was read at 492 nm. Clones with OD values in BLS-Stx2B coated well ≥2 times OD values in BLS coated well, were selected for sequencing analysis.

### Production and formatting of VHHs

VHHs genes were amplified by PCR and were sub-cloned in pHEN6 vector^44^. *E. coli* WK6 electrocompetent cells were transformed with pHEN6 vector containing VHH genes. Expression was induced by addition of IPTG to final concentration of 1 mM and incubated over night at 28 °C with shaking. The periplasmic fraction was obtained, dialyzed and injected to a HisTrap HP column. Elution was performed with 50 mM NaH_2_PO_4_, 300 mM NaCl, 500 mM imidazole, pH 8.

The selected Stx2B-specific 2vb27 VHH was converted into a bivalent and a trivalent format joining each VHH through the linker sequence (Gly_4_-Ser)_3_^45^. Nucleotide sequences for fusion proteins were obtained from GenScript (GenScript, Piscataway, NY). The bivalent format [(2vb27)_2_] was generated by fusion of two 2vb27 copies. For the trivalent format [(2vb27)_2_-SA], a VHH with affinity for human and mouse serum albumin (SA)^28^ was attached to two copies of 2vb27, one through its N-terminal end and other through its C-terminal end. Both molecules were cloned in pHEN6 vector and purified with HisTrap HP as mentioned above.

### *In vitro* Stx2-neutralization assay on Vero cells

Recombinant Stx2 (rStx2) was expressed and purified as previously described^46^. Serially diluted purified 2vb27, (2vb27)_2_ or (2vb27)_2_-SA were pre-incubated with 1CD_50_ (9.5 fmoles of Stx2, cytotoxic dose that kills 50% of Vero cells) of rStx2 for 1 h at 37 °C followed by 1 h at 4 °C. The mixtures were overlaid to each well containing 10^4^ Vero cells and incubated for 48 h at 37 °C in 5% CO_2_. Cells were washed with PBS, stained with crystal violet dye (0.1% crystal violet in 20% methanol:water solution) and read on a microtiter plate reader (Asys UVM340; Biochrom Ltd., Cambridge, England) with a 570 nm filter. The percentage of cell survival, which is a measurement of the toxin neutralization, was calculated by the following formula: [(OD_toxin+VHH_ − OD_toxin only_)/(OD_no toxin_ − OD_toxin only_)] × 100. The fifty percent inhibitory concentration (IC_50_) was determined as the VHH concentration that produced a signal that was 50% of the difference between the maximum response and the baseline signal from wells having no VHH.

### Human SA-binding ELISA assay

Pierce^®^ Nickel coated plates were blocked with 0.5% BSA-PBS and incubated with serially diluted (2vb27)_2_-SA in 0.5% BSA-PBS. Biotinylated human SA (abcam^®^, Ltd., UK; 12.5 ng/well) was added and binding was detected with streptavidin-HRP diluted 1:8000 (Thermo, Cleveland, OH). Reaction was developed with 3,3′,3,5′-Tetramethylbenzidine (TMB; Invitrogen) and absorbance was read at 450 nm.

### *In vivo* half-life determination

Naïve adult BALB/c mice (4 mice/group) were intravenously (i.v.) inoculated with 0.5 nmoles of 2vb27, (2vb27)_2_ or (2vb27)_2_-SA. Animals were bled at 5 min, 1 h, 5 h, 1 day, 7 days or 15 days. Remaining VHH in plasma was determined by *in vitro* Stx2-neutralizing activity as described above.

### *In vivo* Stx2- neutralizing capacity

Three different models of Stx2-dependent toxicity were used to test *in vivo* Stx2-neutralization activity and protective capacity. A) Unique high dose model: naïve adult BALB/c mice were injected i.v. with a unique dose of 0.05 pmoles/mouse of rStx2, which represents a lethal dose 100% (1LD_100_, dose that kills 100% of mice). VHH in their different formats were i.v. administered simultaneously with rStx2 (3–6 mice/group were used). B) Incremental split–dose model: The lethal dose of rStx2 (0.05 pmoles/mouse) was divided in four doses that were i.v. administrated to naïve adult BALB/c mice once a day for 4 consecutive days. The first two doses were lower (0.009 pmoles/mouse each dose) and the last two doses were higher (0.016 pmoles/mouse each dose). To determine the therapeutic window, 4–6 mice/group were used. To determine the lowest protective dose of (2vb27)_2_-SA, 3–15 mice/group were used. C) STEC intragastric infection model: Immature male and female BALB/c mice (5–6 mice/group) were used immediately after weaning (17–19 days of age, ~8–11 g of body weight). Weaned mice were divided randomly into experimental groups. After 8 h of starvation, animals were intraperitoneally (i.p) inoculated with 100 μl of PBS or (2vb27)_2_-SA and intragastrically (i.g.) inoculated via a stainless steel canulae (model 7.7.1, 0.38 mm × 22 G) (Harvard Apparatus, Holliston, MA) with 0.1 ml of Stx2-producing *E. coli* O157:H7 (referred as STEC) bacterial suspension (4 × 10^11^ CFU/kg). STEC was isolated from a fecal specimen of a child with HUS and bacterial cultures were performed as previously described by Brando *et al.*^32^. Food and water were provided to mice ad libitum 4 h after the ingestion of the bacterial suspension.

For all models, mice were monitored three times a day and those becoming moribund were humanely euthanized by CO_2_ inhalation in a closed chamber.

### Clinical assessments

Blood samples were obtained for laboratory analyses that included total and differential blood cell count in Neubauer chamber and blood urea nitrogen determination. Biochemical determinations of urea in mouse plasma were performed with a commercial kit (Wierner Lab, Argentina).

### Evaluation of systemic reticuloendothelial system by antibody-dependent platelet clearance

Liposome-encapsulated clodronate (dichloromethylene bisphosphonate; Lip-Clod) was generously provided by clodronateliposomes.com (Science Park, Amsterdam, Nehterlands). The intravenous injection of 0.1 ml/10 g body weight of this Lip-Clod suspension induces the complete depletion of splenic and hepatic macrophages within 24 h^47^. Adult BALB/c mice (3 mice/group) were i.v. injected with a unique dose of 200 μl of Lip-Clod or Lip-PBS. Twenty four hours later, mice were i.v. injected with 1LD_100_ of Stx2 pre-incubated with or without (2vb27)_2_-SA. For control of Lip-clod efficacy, another group of animals (3–4 mice/group) was injected i.p. with 25 μl of a rabbit anti-mouse platelet antiserum prepared as previously described^48^. Five hours after antiserum inoculation, mice were bled and platelets were counted in a veterinary automatic hematology analyzer (Abacus JuniorVet 5) using a buffer containing ammonium oxalate 1% for erythrocyte lysis.

### Statistical analysis

Data are presented as the mean ± SEM of each group of mice. Statistical differences were determined using one-way Multiple Comparison analysis of variance by ANOVA-Newman-Keuls Test, and p < 0.05 was considered significant. Comparisons between two groups were performed with the Student t test. The Log-rank test was used to compare survival curves. Values of p < 0.05 were considered to be significant.

## Additional Information

**How to cite this article**: Mejías, M. P. *et al.* Development of camelid single chain antibodies against Shiga toxin type 2 (Stx2) with therapeutic potential against Hemolytic Uremic Syndrome (HUS). *Sci. Rep.* **6**, 24913; doi: 10.1038/srep24913 (2016).

## Supplementary Material

Supplementary Information
