# Supplementary material for: Development of camelid single chain antibodies against Shiga toxin type 2 (Stx2) with therapeutic potential against Hemolytic Uremic Syndrome (HUS)
Source: Sci Rep. 2016 Apr 27;6:24913. doi: 10.1038/srep24913 (PMC4847011; doi:10.1038/srep24913)
Supplement: Supplementary Information [file srep24913-s1.pdf]

Development of camelid single chain antibodies against Shiga toxin type 2 (Stx2) with therapeutic potential against Hemolytic Uremic Syndrome (HUS)

Maria P. Mejías; Yanina Hiriart; Constanza Lauché; Romina J. Fernández-Brando; Romina Pardo; Andrea Bruballa; María V. Ramos; Fernando A. Goldbaum; Marina S. Palermo; Vanesa Zylberman.

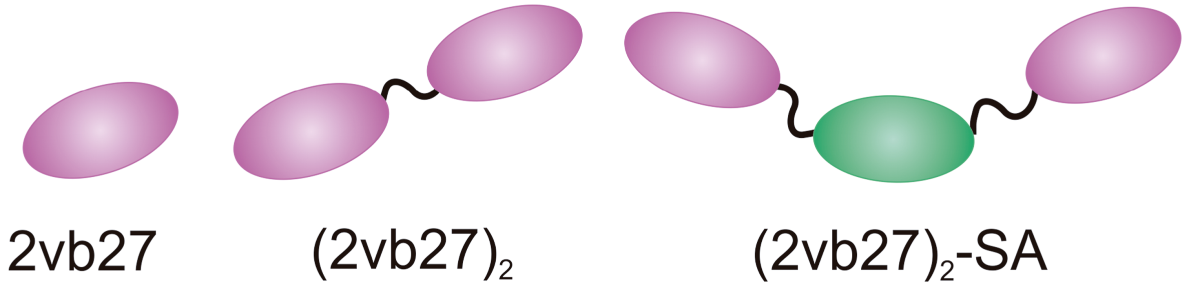

Figure S1. Schematic representation of the different VHH 2vb27 formats.

The selected Stx2B-specific VHH 2vb27 was converted into a bivalent and a trivalent format joining each VHH through the linker sequence (Gly4-Ser)3. Bivalent (2vb27)2 consists of two copies of 2vb27 attached through a 15aa linker. Trivalent (2vb27)2-SA consists of two copies of VHH 2vb27 attached through a 15aa linker to a VHH with affinity for human and mouse serum albumin. VHH 2vb27 is magenta and anti-albumin VHH is green.

Development of camelid single chain antibodies against Shiga toxin type 2 (Stx2) with therapeutic potential against Hemolytic Uremic Syndrome (HUS)

Maria P. Mejías; Yanina Hiriart; Constanza Lauché; Romina J. Fernández-Brando; Romina Pardo; Andrea Bruballa; María V. Ramos; Fernando A. Goldbaum; Marina S. Palermo; Vanesa Zylberman.

		FR1	CDR1	FR2	CDR2	FR3	CDR3	FR4
**Family 1**	**2VB27**	QVQLQESGGGLVQPGGSLKLACAAS	GIT-FRNKAIG	WYRQAPGKGRELVA	RIDSFDTT-DYADSVKG	RFSISRDNAKNTVYLQMNSLKSEDTAVYYC	NLRGSNY	WGQGTQVTVSS
**Family 2**	**2VB23**	QVQLQESGGGLVQAGGSLRLSCTAS	GSI-FNTATMA	WSRQAPGKQRELVA	SITQGRIT-YPVDSVKG	RFTLSRDNSKNTVYLQMNSLEPEDTAVYYC	GVDTIPTSRPRY	WGQGTQVTVSS
**Family 3**	**2VB43**	QVQLQESGGGLVQAGGSLRLSCAAS	ENP-SSISTMA	WYRQAPGKQRELVA	RIITGGYT-NYLDSVMG	RFTISRGNRESTAYLQMNSLKPEDTAVYIC	NARTWSSADY	WGQGTQVTVSS
**Family 4**	**1VB42**	QVQLQESGGGLVQAGGSLRLSCAVS	GRTGNIYAAMG	WFRQAPGKQREFVS	ADSWNAGTTDYADSVKG	RFTISRDNAKSTVYLQMNSLKPEDTAVYYC	AAKIGLYDTSRGRFENEYDY	WGQGTQVTVSS
**Family 5**	**1VB23**	QVQLQESGGGLAQAGGSLRLSCAAS	GFD-FDYYAIG	WFRQAPGKEREGVA	CITDSDGSTIYADSVRG	RFTITADNAENTVYLQMNSLKPEDTAEYFC	AAECFACSGYACHS	WGRGTQVTVSS
**Family 6**	**2VB20**	QVQLQESGGGLVQPGGSLRLSCAAS	GFT-IDYYAIA	WFRQAPGEEREWVS	CIRSGDGSTWYVDSVKG	RFSISSDNAKNAVYLQISSLKPEDTAVYYC	AASRGSPYCPAVIDYDY	WGQGTQVTVSS
**Family 7**	**2VB6**	QVQLQESGGGLVQPGGSLRLSCAAS	GII-FRSKSVG	WYRQAPGTQREWVA	YIS-GDDSTNYEDFVKG	RFTISRDNAKNTVYLQMNSVKPEDTAIYYC	AADYRDYDELLLPVPPPYDY	WGQGTQVTVSS

Table S1: Amino acid sequences from a representative clon from each VHH family.

Clones with Stx2-binding capacity were sequenced and grouped into 7 families based on amino acid composition and length of the CDR3. Sequences show all the regions of the antibody structure. Framework regions (FR1 through FR4) maintain the tertiary structure of the paratope while the three complementarity determining regions (CDR1 through CDR3) form the hypervariable loops that directly interact with the epitope of the antigen.
